# Bilateral renal infarction unmasking rheumatic mitral valve disease with left atrial appendage thrombus in sinus rhythm: a case report

**DOI:** 10.1093/ehjcr/ytag821

**Published:** 2026-10-31

**Authors:** Chia Ching Chen, Tzu-Chen Lu, Yu-Ling Wang, Ya-Lei Niu, Hui-Ching Hung

**Affiliations:** Department of Emergency Medicine, Chang Bing Show Chwan Memorial Hospital, No. 6, Lugong Road, Lukang Township, Changhua County 505, Taiwan; Asian Institute of TeleSurgery (IRCAD-Taiwan), Chang Bing Show Chwan Memorial Hospital, No. 6, Lugong Road, Lukang Township, Changhua County 505, Taiwan; Department of Emergency Medicine, Chang Bing Show Chwan Memorial Hospital, No. 6, Lugong Road, Lukang Township, Changhua County 505, Taiwan; Department of Gastroenterology, Chang Bing Show Chwan Memorial Hospital, No. 6, Lugong Road, Lukang Township, Changhua County 505, Taiwan; Department of Cardiology, Chang Bing Show Chwan Memorial Hospital, No. 6, Lugong Road, Lukang Township, Changhua County 505, Taiwan; Department of Nephrology, Chang Bing Show Chwan Memorial Hospital, No. 6, Lugong Road, Lukang Township, Changhua County 505, Taiwan

**Keywords:** Case report, Renal infarction, Contrast-enhanced ultrasound, Rheumatic mitral stenosis, Left atrial appendage thrombus, Cardioembolism

## Abstract

**Background:**

Acute renal infarction mimics pyelonephritis and is frequently missed at first presentation. Rheumatic mitral stenosis can cause systemic embolism even in sinus rhythm.

**Case summary:**

A 47-year-old Thai man presented with four days of evolving abdominal pain, previously attributed to gastroenteritis and treated symptomatically at a first emergency visit. He had fever without chills, left costovertebral-angle tenderness, and a bland urinalysis. Point-of-care ultrasound was atypical for pyelonephritis; computed tomography showed bilateral wedge-shaped cortical defects with a cortical rim sign but could not exclude focal bacterial nephritis. Contrast-enhanced ultrasound demonstrated an avascular left upper pole, confirming bland infarction, supported by lactate dehydrogenase 1194 U/L, procalcitonin 0.28 ng/mL, and sterile cultures. Echocardiography revealed rheumatic mitral valve disease with clinically significant stenosis (valve area 1.34 cm^2^ by planimetry; diastolic doming, commissural fusion, fish-mouth orifice) and moderate regurgitation. The left atrium was dilated, with dense spontaneous echo contrast and a left atrial appendage thrombus despite sinus rhythm. Enoxaparin with overlapping warfarin was started; a direct oral anticoagulant was avoided. At 2 months the patient was in New York Heart Association class II without recurrent embolism, but the international normalized ratio had not yet reached target; repeat transoesophageal echocardiography is scheduled at 3 months.

**Discussion:**

Contrast-enhanced ultrasound can resolve the computed tomography ambiguity between infarction and focal bacterial nephritis at the bedside. In mitral stenosis, a prior embolic event or left atrial thrombus mandates vitamin K antagonist anticoagulation irrespective of rhythm, and an appendage thrombus and coexisting mitral regurgitation defer balloon commissurotomy.

Learning points

- Rheumatic mitral stenosis can embolize in sinus rhythm; a left atrial appendage thrombus or a prior embolic event mandates vitamin K antagonist therapy and defers balloon commissurotomy.
- Contrast-enhanced ultrasound separates renal infarction from focal bacterial nephritis at the bedside when computed tomography is equivocal, without radiation or nephrotoxic contrast.

## Introduction

Acute renal infarction is rare, is frequently mistaken for pyelonephritis or renal colic, and is often diagnosed late.^1,2^ Cardioembolism is the leading mechanism; rheumatic mitral stenosis remains an important source in endemic regions and can embolize in sinus rhythm through left atrial stasis. Computed tomography usually confirms the diagnosis but may not separate infarction from focal bacterial nephritis. Contrast-enhanced ultrasound demonstrates absent perfusion at the bedside without radiation.^3,4^ We describe a nurse practitioner-directed pathway to this diagnosis.

## Summary figure

**Figure:**
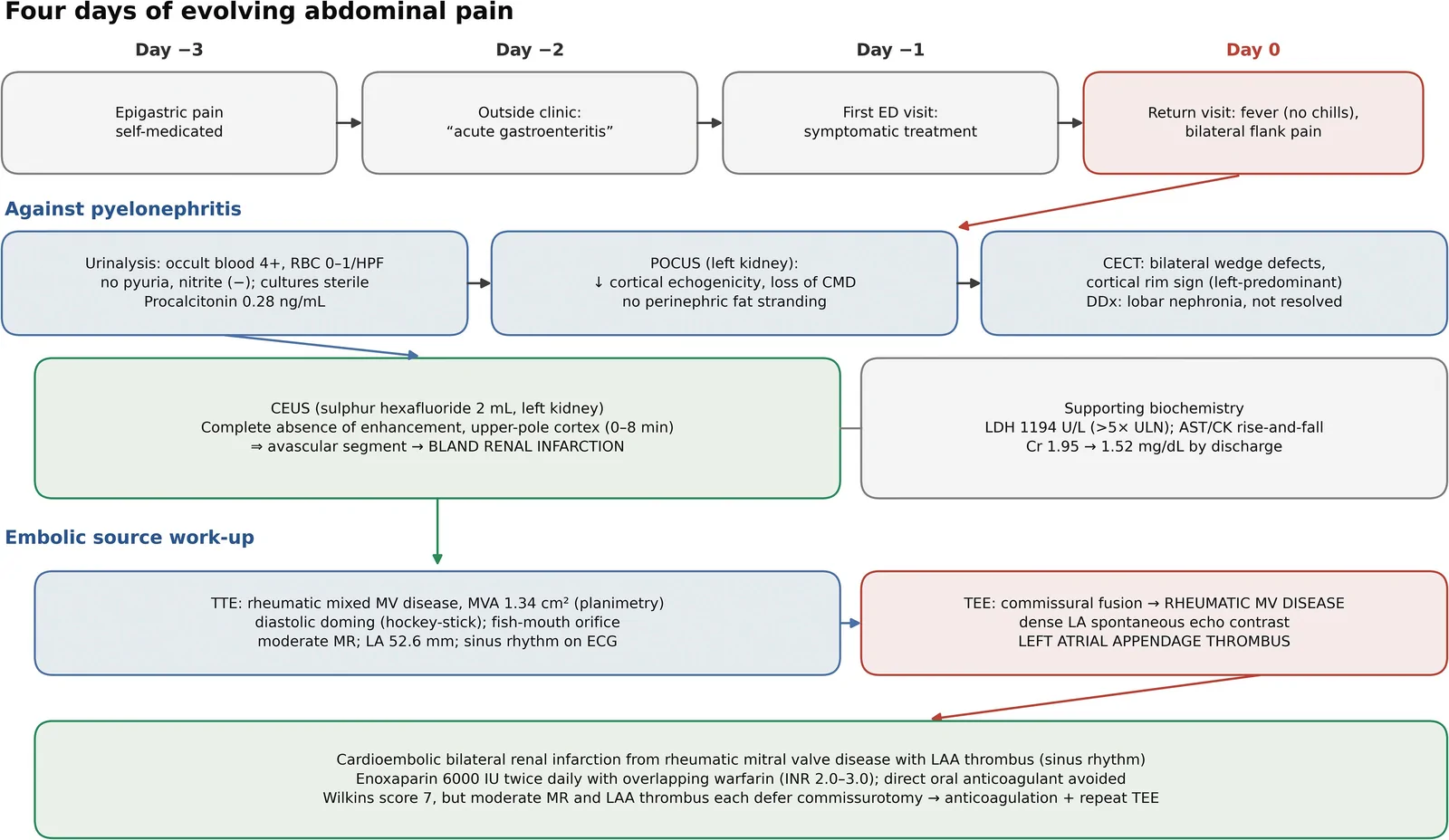
Diagnostic pathway from non-specific abdominal pain to cardioembolic bilateral renal infarction, with key investigations and decision points.

## Case presentation

A 47-year-old Thai man presented with 4 days of evolving abdominal pain, attributed to gastroenteritis at an outside clinic and at a first emergency department visit. By presentation, the pain was generalized with bilateral flank pain, cold sweating, and fever without chills. He drank approximately six bottles of beer daily for 7 years and smoked half a pack daily. There was no known hypertension, diabetes, atrial fibrillation, or thromboembolism, and childhood rheumatic fever could not be ascertained.

Temperature was 38.5°C, heart rate 90 beats per minute, blood pressure 118/76 mmHg, and oxygen saturation 97%. The abdomen was soft with epigastric and left costovertebral-angle tenderness; no diastolic murmur was appreciated.

Laboratory studies showed leucocytosis (19 830/µL, 82% neutrophils), C-reactive protein 9.83 mg/dL, and acute kidney injury (creatinine 1.95 mg/dL, estimated glomerular filtration rate 42 mL/min/1.73 m^2^). Urinalysis was bland: dipstick occult blood 4+ with 0–1 red cells per high-power field, negative leukocyte esterase and nitrite, no pyuria, and a sterile culture. Lactate dehydrogenase was 1194 U/L; aspartate aminotransferase and creatine kinase fell from 181 to 78 U/L and from 336 to 137 U/L, respectively; procalcitonin was 0.28 ng/mL, and blood cultures were sterile.

Point-of-care ultrasound showed an enlarged left kidney with a hypoechoic upper pole and loss of corticomedullary differentiation, without perinephric stranding (*Figure 1A*). Contrast-enhanced computed tomography showed multiple wedge-shaped low-density lesions in both kidneys with a cortical rim sign, left-predominant; lobar nephronia remained a differential because dynamic phases were not performed (*Figure 2*). Contrast-enhanced ultrasound of the left kidney (2 mL sulfur hexafluoride microbubbles) showed no enhancement of the upper-pole cortex, apart from a thin capsular rim, from injection through eight minutes, confirming bland infarction (*Figure 1B*, Supplementary material online, *Video S1*). The small right-sided lesions were seen on computed tomography only.

**Figure 1:**
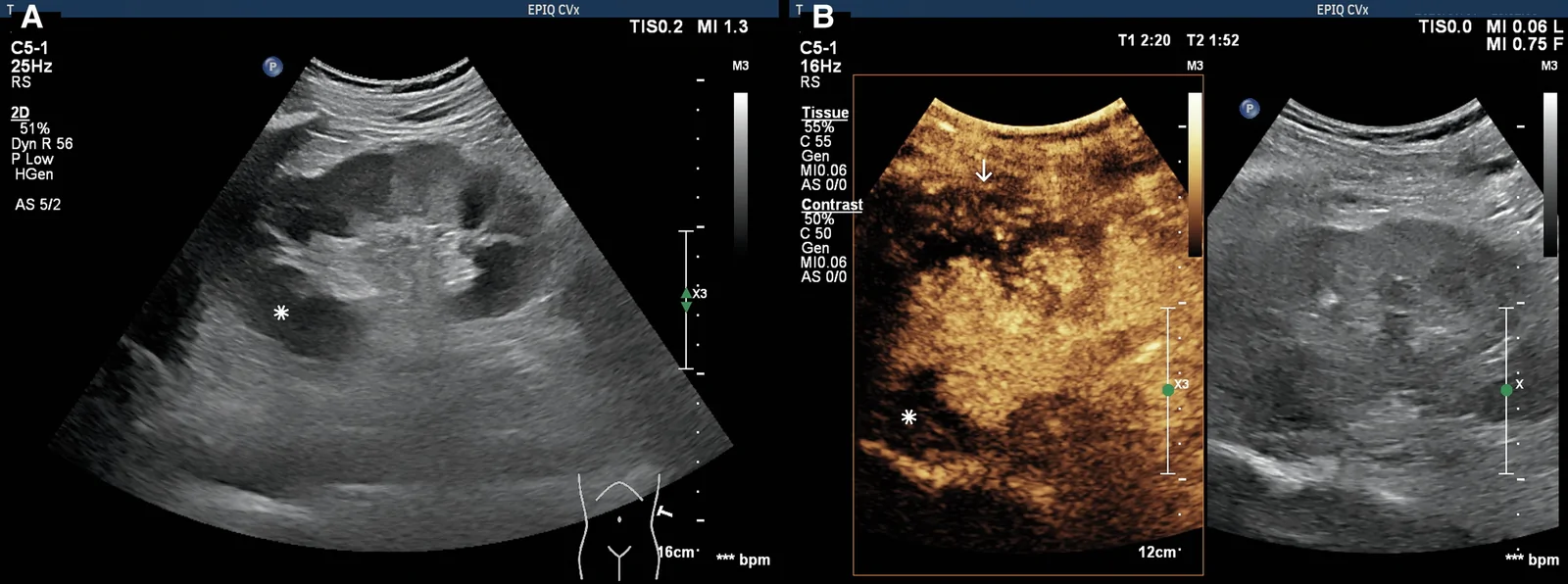
Left kidney ultrasound. (*A*) Greyscale image showing decreased cortical echogenicity and loss of corticomedullary differentiation at the upper pole (asterisk). (*B*) Contrast-enhanced ultrasound (sulfur hexafluoride microbubbles) with side-by-side contrast and tissue display showing absent enhancement of the upper-pole cortex (asterisk), apart from a thin enhancing cortical rim (arrow), the cortical rim sign, confirming an avascular segment.

**Figure 2:**
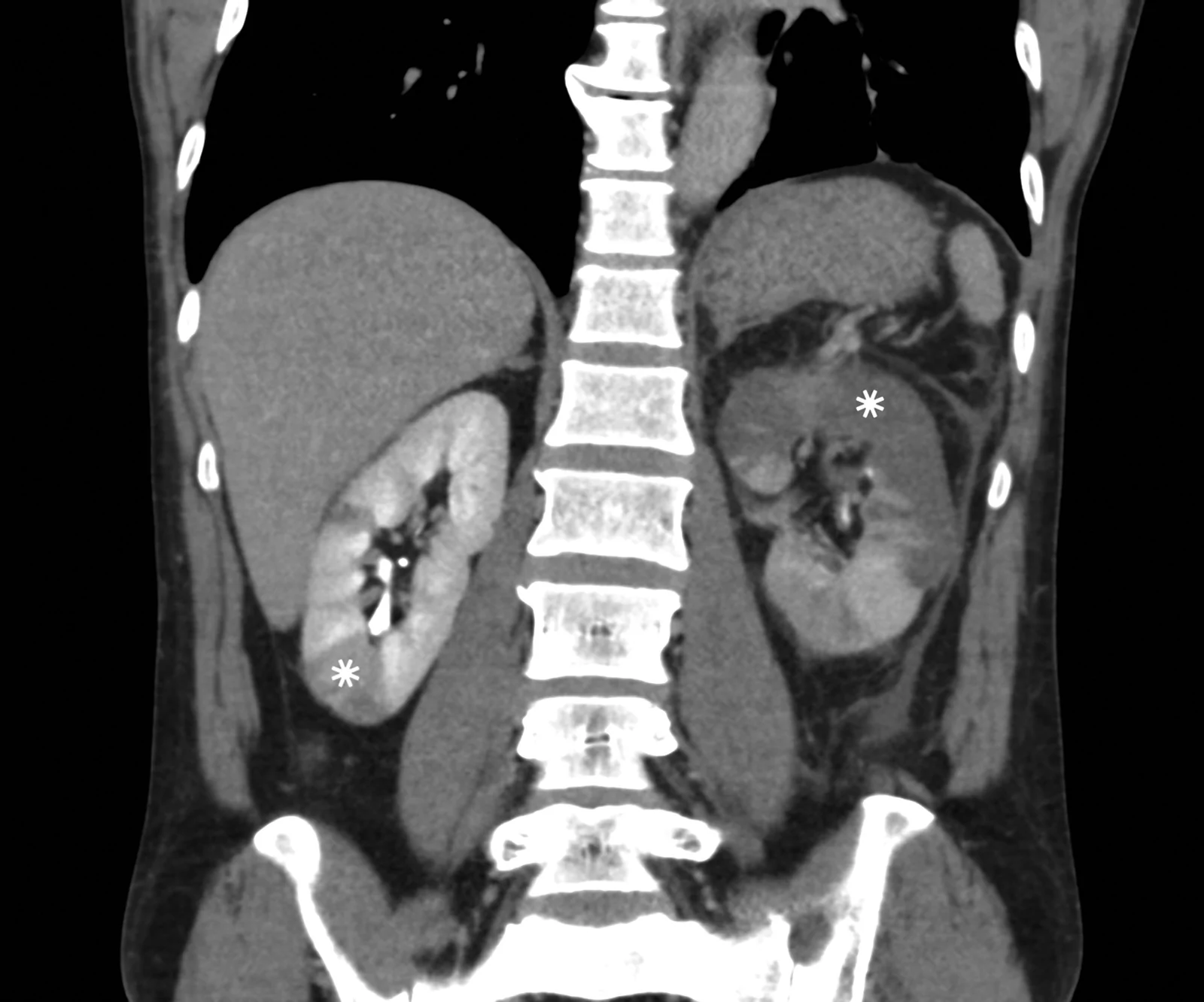
Contrast-enhanced abdominal computed tomography showing multiple wedge-shaped cortical low-density lesions with absent parenchymal enhancement in both kidneys (asterisks), left-predominant, with a cortical rim sign, suggestive of renal infarction.

Electrocardiography showed sinus rhythm. Transthoracic echocardiography showed rheumatic mixed mitral valve disease. The valve area was 1.34 cm^2^ by two-dimensional planimetry; the pressure half-time estimate (1.96 cm^2^) was discounted because reduced left ventricular compliance (E/e′ 15.6) shortens the half-time. The mean transmitral gradient was 5 mmHg at 58 beats per minute. The anterior leaflet showed diastolic doming (hockey-stick sign), the short-axis view a fish-mouth orifice, and three-dimensional en-face imaging a slit-like orifice (*Figure 3*). Mitral regurgitation had a vena contracta of 5.1 mm in the moderate range; there were aortic regurgitation and moderate tricuspid regurgitation with severe pulmonary hypertension (peak tricuspid velocity 4.0 m/s; estimated pulmonary artery systolic pressure ∼72 mmHg). The left atrium was dilated (anteroposterior diameter 52.6 mm; volume index 80 mL/m^2^) and left ventricular systolic function was preserved. Transoesophageal echocardiography confirmed commissural fusion, with dense spontaneous echo contrast throughout the left atrium and a left atrial appendage thrombus (*Figure 4*, Supplementary material online, *Videos S2*–S3). The Wilkins score was 7 (leaflet mobility 2, thickening 3, subvalvular thickening 1, and calcification 1).

**Figure 3:**
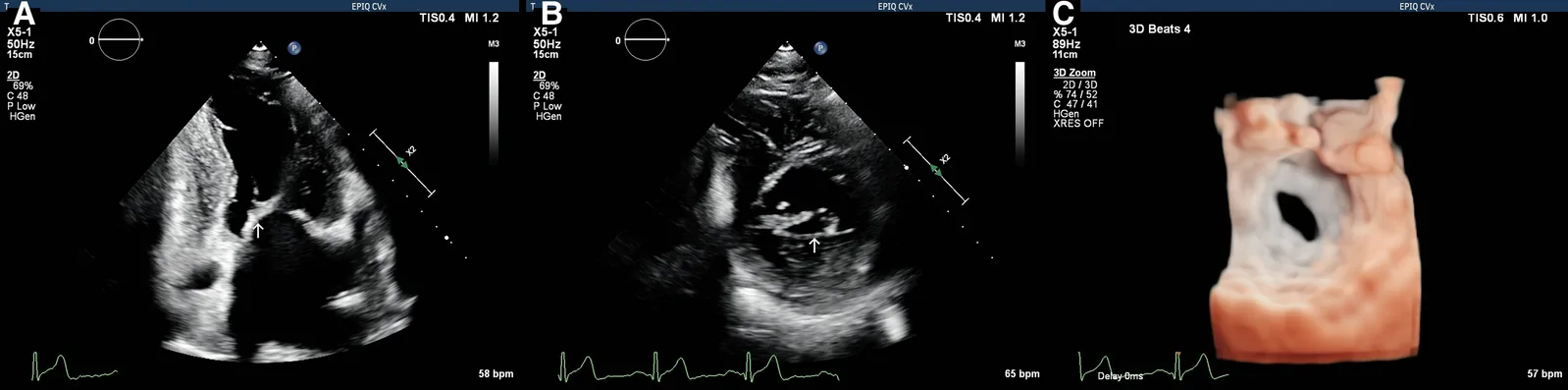
Transthoracic echocardiography. (*A*) Apical view showing diastolic doming of the anterior mitral leaflet, the hockey-stick sign (arrow). (*B*) Short-axis view at the mitral level showing a fish-mouth (buttonhole) orifice from commissural fusion (arrow). (*C*) Three-dimensional en-face view of the mitral valve from the left atrium in diastole showing the slit-like rheumatic orifice with thickened leaflets.

**Figure 4:**
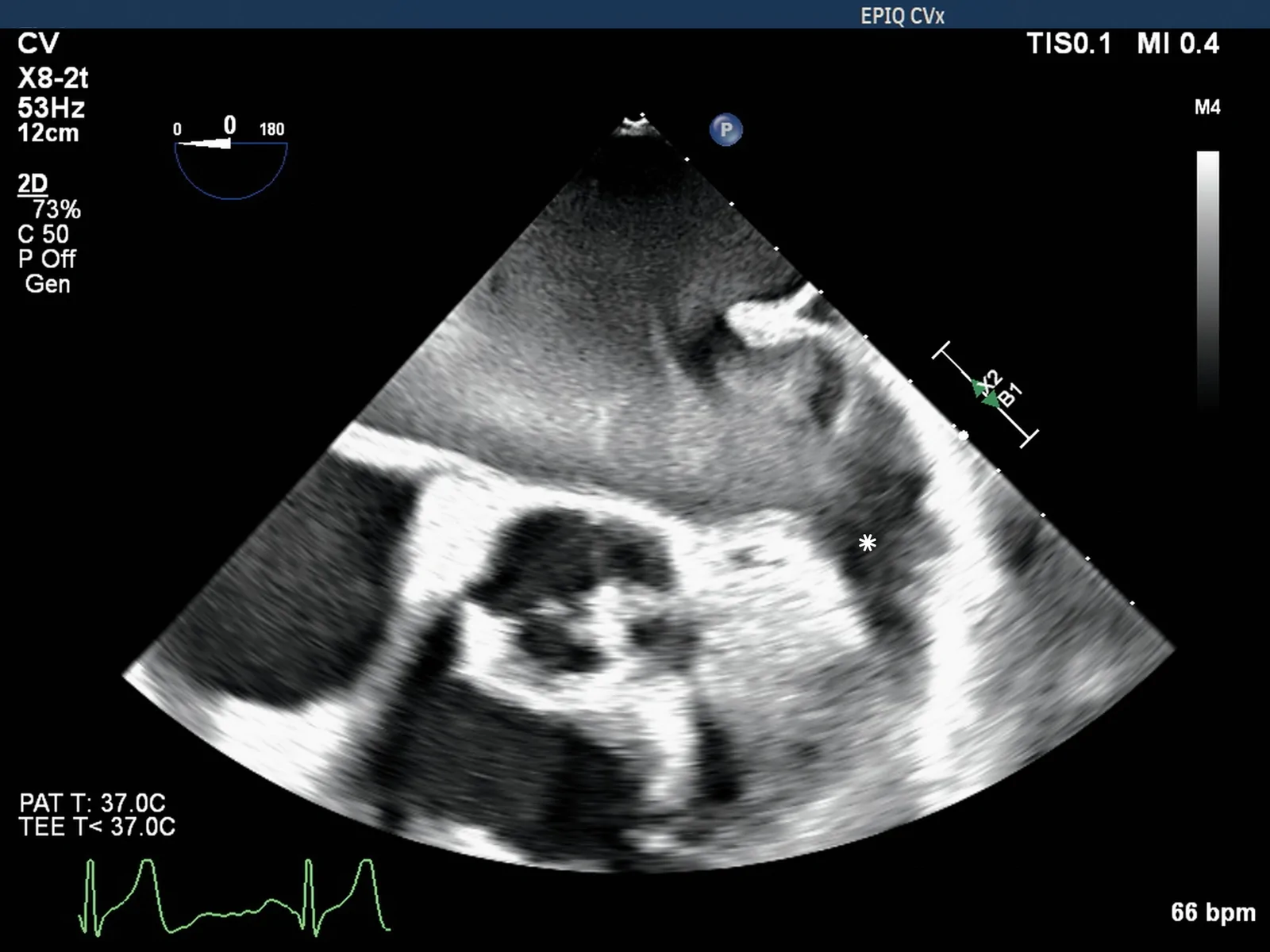
Transoesophageal echocardiography showing dense spontaneous echo contrast within the left atrium and a left atrial appendage thrombus (asterisk).

Enoxaparin 6000 IU twice daily was started with overlapping warfarin (target international normalized ratio 2.0–3.0); a direct oral anticoagulant was avoided. By discharge, creatinine had improved from 1.95 to 1.52 mg/dL and white cells from 19 830 to 12 490/µL; he was discharged on warfarin alone.

At 2 months (clinic visit 8 September 2026), he was in New York Heart Association class II with no recurrent embolic event; serum creatinine was 1.79 mg/dL on 14 August 2026. On warfarin 2.5 mg daily, none of four international normalized ratio determinations (1.05, 1.02, 1.12, and 1.14) reached the 2.0–3.0 target, and the dose was increased to 5 mg daily. Repeat transoesophageal echocardiography was scheduled for 8 October 2026.

## Discussion

This case carries two messages. The first is that contrast-enhanced ultrasound can resolve the ambiguity that computed tomography leaves open. Renal infarction belongs on the differential when flank pain and fever come with a urinalysis without pyuria and a disproportionately raised lactate dehydrogenase.^1,2^ Dynamic computed tomography phases might have settled the question, but the patient had acute kidney injury; microbubbles are not nephrotoxic. Lobar nephronia retains perfusion, albeit heterogeneous,^3,4^ and an abscess shows a thick enhancing wall. Here the non-enhancing area was wedge-shaped and cortical with only a thin capsular rim; with low procalcitonin and sterile cultures, this excluded an infective process.

The second is that rheumatic mitral stenosis can embolize in sinus rhythm. Atrial stasis, rather than the rhythm, generates the thrombus. A markedly dilated left atrium, dense spontaneous echo contrast, and an appendage thrombus were present in sinus rhythm, and the bilateral, multifocal infarcts favoured a central source. Left atrial thrombus occurs in about 7% of mitral stenosis patients in sinus rhythm,^5^ clusters with atrial enlargement and dense spontaneous echo contrast,^5,6^ and is unlikely when contrast is absent.^5^ European guidance accordingly advises considering anticoagulation in sinus rhythm when dense spontaneous echo contrast or a left atrial volume above 60 mL/m^2^ is present, even without thrombus.^7^ Valve area, gradient (at only 58 beats per minute), atrial volume, and pulmonary pressure were concordant, the pressure half-time estimate having been discounted as above. The thrombus and the embolic event each constitute a Class I indication for vitamin K antagonist anticoagulation irrespective of rhythm.^7,8^ For rheumatic mitral stenosis with atrial fibrillation, European guidance recommends vitamin K antagonists rather than direct oral anticoagulants,^7,9^ and the INVICTUS trial showed rivaroxaban inferior to vitamin K antagonist therapy in rheumatic heart disease with atrial fibrillation.^10^ Neither addresses sinus rhythm, and direct-oral-anticoagulant data for appendage thrombus derive from non-valvular populations; because the stasis physiology here matches the trial population, a vitamin K antagonist was the conservative extrapolation.

Percutaneous mitral commissurotomy was deferred rather than rejected. The Wilkins score of 7 indicates leaflet morphology suitable for the procedure.^11^ Left atrial thrombus, here in the appendage, is an absolute contraindication, because catheter manipulation across the left atrium risks dislodging it. Mitral regurgitation greater than mild (here moderate by vena contracta) is a further contraindication, because commissurotomy characteristically worsens regurgitation.^7^ The plan was three months of vitamin K antagonist therapy (international normalized ratio 2.0–3.0), then repeat transoesophageal echocardiography to determine whether the thrombus had resolved. Should it resolve while regurgitation remains moderate, commissurotomy would remain contraindicated and, with class II symptoms and a valve area less than 1.5 cm^2^, surgical valve replacement would be considered.^7^ The early anticoagulation record is itself a lesson. Enoxaparin was not continued after discharge, and the dose was first adjusted at the 4-week outpatient visit; in a patient with heavy alcohol use, earlier and more frequent monitoring was warranted.

Paroxysmal atrial fibrillation before presentation cannot be excluded. Telemetry was continuous during admission, but no ambulatory monitoring was performed after discharge. The anticoagulation indication, however, rests on the thrombus and the embolic event and would be unchanged. Endocarditis was unlikely given sterile cultures, low procalcitonin, and no vegetations on transoesophageal echocardiography. Mitral regurgitation was graded by vena contracta alone.

## Lead author biography


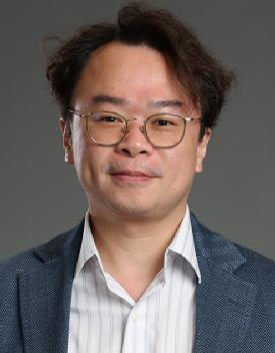


Chia Ching Chen, MD, is an attending emergency physician at Chang Bing Show Chwan Memorial Hospital, Changhua, Taiwan, where he directs the Ultrasound Training Center of the Show Chwan Healthcare System and the Emergent Transoesophageal Echocardiography Resuscitation Center. He is a WINFOCUS-certified instructor, faculty of IRCAD-Taiwan/AITS, a member of the academic committee of the Society of Ultrasound in Medicine of the Republic of China, and medical director of the Changhua County Fire Bureau. His interests include emergency point-of-care ultrasound, resuscitative transoesophageal echocardiography, and prehospital ultrasound.

## Supplementary Material

ytag821_Supplementary_Data

## Data Availability

The data underlying this case report are presented within the article and its Supplementary material online, *Videos*. Further anonymised data are available from the corresponding author on reasonable request, subject to institutional and patient-privacy restrictions.
